# Assessment of factors affecting the sleep hygiene of medical students in Bahawalpur, Pakistan: a cross-sectional study

**DOI:** 10.5935/1984-0063.20200063

**Published:** 2021

**Authors:** Zarafshan Shaﬁque, Faiza Syed, Saﬁa Naz, Saba Urooj, Sadia Khan, Sabahat Javed

**Affiliations:** Quaid-e-Azam Medical College, Department of Community Medicine - Bahawalpur - Punjab - Pakistan.

**Keywords:** Sleep Hygiene, Medical Students, Sleep Quality, Sleep Disturbance

## Abstract

**Objective:**

To identify the factors which have a statistically signiﬁcant association with sleep hygiene of medical students.

**Material and Methods:**

This study was done on 100 medical students. The questionnaire that was used for the collection of data had two parts. First was related to demographic variables and second was a validated Pittsburgh sleep quality index (PSQI). All participants were from the ﬁrst to ﬁfth years of Bachelor of Medicine and Bachelor of Surgery (MBBS). Data were analysed using SPSS 23. The association was found by applying chi-square test.

**Results:**

Our study revealed that out of 100 students, 61% had poor sleep hygiene (PSQI ≥ 5). Global PSQI scores among women were slightly higher than males. A statistical association was found between the residence and habit of studying at night of medical students with their sleep hygiene.

**Discussion:**

Medical students should ensure good sleep hygiene in order to maintain their academic performance, physical health, and mental health.

## INTRODUCTION

Sleep is defined as a natural state of rest during which the subject is unconscious but can be awaken with the help of proper external stimuli^1^. It is well known that a good quality and adequate amount of sleep are basic human necessities. According to sleep specialists, most healthy young adults and adults require at least seven to nine hours of sleep per day for optimal health and functioning of their mind and bodies^2^.

Sleep hygiene refers to those behaviours that ensure an improved quality and quantity of sleep to a person on a regular basis^1^. Reports show that about one-third of adults of the general population have some form of insomnia. Being a subgroup of the general population medical students are at an increased risk of developing a poor sleep hygiene, which, is mostly attributed to their challenging academic and clinical duties, long duration of study hours, stress of examination, hectic routine, and lifestyle choices^3^. Excessive use of caffeine, alcohol or energy drinks and unrestrained use of mobile phones, tablets or laptops before sleep are also some of those common factors that prevent maintaining good sleep hygiene^4^.

Most of the medical students do not give much importance to their sleep as they think that reducing the sleeping hours will help them cope with the piles of academic work that needs to be done in a short period of time. As a result, they develop poor sleeping habits in the weeks before the commencement of their examination and these altered sleeping patterns persist even after the examination is over^5^.

Studies have shown that deprivation of sleep results in impaired consciousness, sleepiness during daytime, lack of attention, compromised relationships, and fatigue^6^. These ill effects may in turn cause the students to commit medical errors and influence the patient safety^7^.

Sleep disturbances in medical students lead to such unwanted consequences that badly affect their academic performance, learning behaviours, and overall health^8^. Therefore, the importance of sleep quality among medical students cannot be overlooked as it has an obvious impact on the mental health, stress levels, and patient care^9^.

The objective of this study was to highlight those factors that have a statistically significant association with sleep hygiene. Since only a limited work has been done in Pakistan regarding this topic, our research will not only help doctors in expanding their knowledge but it will also serve to signify the importance of this matter in an attempt to improve the sleep hygiene of medical students across the country.

## MATERIAL AND METHODS

This cross-sectional study was conducted at Quaid-e-Azam Medical College, Bahawalpur after approval from the ethical review committee. A total of 100 medical students were recruited who were willing to participate in the study. The duration of study was from March 2020 to June 2020. The data were collected face-to-face using a self-administered questionnaire, which comprised of two parts (Questionnaire 1). Part one had seven questions related to demographic variables and part two was a preformed pretested Pittsburgh sleep quality index (PSQI), which is a great tool for the evaluation of sleep hygiene during the past month. It consists of 19 items, which when combined yield seven sub-scales. Scoring of each item is done on a 3 point scale. The combination of these seven sub-scales produces a global PSQI score whose value ranges from 0 to 21. A global PSQI score of less than five indicated ‘good’ sleep hygiene in that subject while a score of equal to or greater than five meant ‘bad’ sleep hygiene^10^.

Data were analysed using SPSS v. 23. Frequencies and percentages were calculated for qualitative variables. The chi-square test was applied to test the significance of association between categorical variables. A *p*-value of less than 0.05 was considered as statistically significant.

The associated factors were exploited using both parts of the applied questionnaire. **Inclusion criteria:** all the students from first to fifth years of Bachelor of Medicine and Bachelor of Surgery (MBBS) who were willing and gave consent to participate in the study. **Exclusion criteria:** The students from first to fifth years of Bachelor of Medicine and Bachelor of Surgery (MBBS) who were not willing to participate and were not available during the study duration.

## RESULTS

In our study, there were 49 males and 51 females. Majority, i.e., 78% was resident of hostel (on-campus). Equal participants (20%) were chosen from each year of MBBS. The mean age of study participants was 21.13±1.824 years.

Out of 100 students, 61% were found to have poor sleep hygiene (their PSQI score was either equal to or more than five). Global PSQI scores among women were slightly higher than males (54.09% of females vs. 45.9% of males). Use of sleep medications was higher in males. Six male students vs. none of the female students used medication to sleep **less than once a week** (this is an idiomatic phrase and it was as such mentioned as one of the options to some questions in the PSQI questionnaire. It practically means that they **did not** use medication **every week**, rather once or twice a **month**). There was no difference in sleep efficiency of both genders.

### Components of Pittsburgh sleep quality index (PSQI)

#### Subjective sleep quality

**Figure 1:**
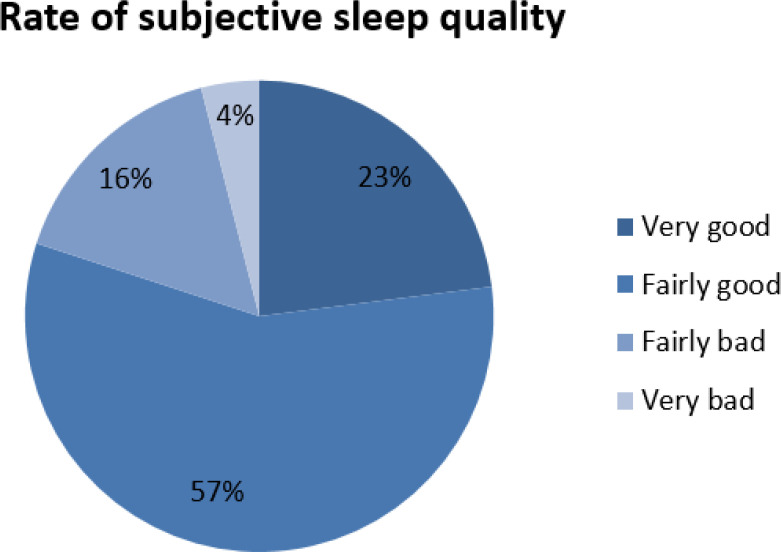
Rate of subjective sleep quality of medical students.

According to Figure 1, 80% students had good sleep while 20% students regarded their sleep quality as bad.

#### Sleep latency

14% of study subjects it took to fall asleep within 15 minutes each night while 84% used to fall asleep after 15 minutes to more than an hour. 46% had trouble getting to sleep within 30 minutes less than once a week.

#### Sleep duration

30% used to spend more than seven hours in bed. 26% spent six to seven hours, 30% spent five to six hours while 14% spent less than five hours in bed.

#### Sleep efficiency

71% of study subjects had sleep efficiency greater than 85%.

#### Sleep disturbance

23% used to wake up in the middle of the night once a week, which disturb their sleep, while 19% had disturbed sleep because of having bad dreams once or twice a week.

#### Daytime dysfunction

Out of 100 students, 41% had trouble staying awake less than once a week while driving, eating meals and engaging in social activities while it had been somewhat of a problem for 30% to keep up enough enthusiasm to get the things done.

#### Use of sleep medications

92% of the students did not use any type of sleep medication during the past month. 6% used sleeping medications less than once a week (**zero times a week but once or twice in the past month**) while only 2% used them once or twice a week.

As shown in Table 1, the p value obtained was 0.029 (which is less than 0.05), which meant that the association between sleep hygiene and the residence of medical students was significant. The students who resided on-campus were more likely to have bad sleep hygiene as compared to those who resided in their homes.

**Table 1.** Association of residence with sleep hygiene.

Residence	Global PSQI score		Total
	< 5	≥5	
On-campus	26	52	78
Off-campus	13	9	22
Total	39	61	100

$\left(\chi^{2}=4.786;\;\mathrm{df}-1;\;p=0.029\right).$

According to Table 2, the calculated *p* value was 0.020, which confirmed that those students who studied late at night actually had bad sleep hygiene as their global PSQI scores were comparatively greater than 5.

**Table 2.** Association of studying at night with sleep hygiene.

Studying at night	Global PSQI score		Total
	< 5	≥5	
Yes	26	52	78
No	13	9	22
Total	39	61	100

$\left(\chi^{2}=5.450;\;\mathrm{df}-1;\;p=0.020\right).$

Sleep hygiene of medical students did not seem to be affected by their gender as shown in Table 3.

**Table 3.** Association of gender with sleep hygiene.

Sleep hygiene	Male	Female	Total
Good sleep	21	18	39
Bad sleep	28	33	61
Total	49	51	100

$\left(\chi^{2}=0.601;\;\mathrm{df}-1;\;p=0.438\right).$

## DISCUSSION

The findings of our study showed that 61% of all students had poor sleep hygiene. And 14% of students got less than 5 hours of sleep per night. In a study conducted by Waqas et al.^11^, on the association of academic stress with sleeping difficulties in medical students in Lahore (Pakistan), it was found that 77% of the respondents were poor sleepers. Academic stressors were a significant cause of poor sleep hygiene in these students. A high percentage, i.e., 27.8% of the total respondents used to get less than 5 hours of sleep per night^11^. An interesting finding of our study is that a statistical association was found between the residence and quality of sleep. The students who lived in hostels had comparatively bad sleep hygiene (85.24%) than those who lived in their homes (14.75%). This is opposite to the findings of a study conducted in West Indian Metropolitan city by Chutani et al.^12^, where more day scholars (73.7%) reported bad sleep quality as compared to hostellers (40.5%).

This could be due to the additional burden of travelling back and forth between college and home that consumes a lot of their time along with the academic load, which prevents them from getting a proper sleep. Hostellers, on the other hand, are provided with living accommodations in the vicinity of their college, so some of their time is saved that they can utilize in making up for their lost sleep due to extracurricular activities and odd sleeping hours^12^.

Our study reported that higher percentage of females (54.09%) had poor sleep hygiene as compared to males (45.9%). Our results were consistent with a study conducted in Dehradun which revealed that 59.3% of females had shown a poor quality of sleep, which was attributed to the long hours of mobile phone usage by them^13^.

No significant association was found between genders and sleep hygiene in our study. This is similar to the results of another study conducted on the evaluation of sleep hygiene among medical students in Saudi Medical College^14^.

In a study conducted by Alsaggaf et al.^15^, on sleep quantity, quality and insomnia symptoms of medical students during clinical years in Kingdom of Saudi Arabia, it was reported that 30% of students had a poor sleep quality. Caffeinated beverages were taken by a high percentage (65%) of students during the day. This finding is consistent with our study where 54% of total students used to take coffee or tea every day (this was asked in first part of the applied Questionnaire 1).

A significant statistical association was found between the consumption of caffeinated drinks and poor sleep quality in a study conducted at King Abdul-Aziz University, Jeddah, KSA while no such statistical association was found in our study^16^.

Another finding of our study was that only a low percentage (6%) of students used to take sleep medications less than once a week and most of them were males. In a study conducted among Sudanese medical students by Mirghani et al.^17^, no difference was found for the use of sleep medications among excellent and average groups of students (23% among excellent group and 22.6% among average group).

There were some limitations in our study. The sample size was very small and it was done in only one institute so the results cannot be generalized. The effect of factors such as stress, anxiety and depression were not studied in this research. Therefore, we propose other researchers to take these points into consideration while conducting a study on sleep hygiene of medical students in future.

## CONCLUSION

In addition to the high prevalence of poor sleepers, our study has been able to find a significant association between the residence at hostel and habit of studying at night with the bad sleep hygiene of medical students. It is very important to arrange educational programs for the medical students in which they should be taught about the measures to improve their sleep hygiene and the ways through which they can fix their routines. Medical students should be assessed for their stress and anxiety profiles by their respective institutions and should be counselled accordingly.

## QUESTIONNAIRE

**Assessment of Sleep Hygiene among Medical Students of Quaid-e-Azam Medical College, Bahawalpur**

Name:____________________________________________________________(optional)

**1. What is your gender? Male / Female**

**2. What is your age?** ________________________________________________________

**3. What is your marital status? Unmarried / Married**

**4. In which year of MBBS are you studying?**

a) 1st b) 2nd c) 3rd d) 4th e) 5th

**5. Where is your residence? On-campus / Off-campus**

**6. How often do you take Coffee/Tea?**

a) Daily b) Weekly c) Never

**7. Do you study late at night? Yes / No**

**Pittsburgh Sleep Quality Index (PSQI)**

**1. During the past month, what time have you usually gone to bed at night?**

a) Before 12:00 am b) 12:00 - 12:59 am c) 01:00 - 01:59 am d) 02:00 - 02:59 am e) After 03:00 am

**2. During the past month, how long (in minutes) has it usually taken you to fall asleep each night?**

a) Within 30 min (3 or more times/ week) b) More than 30 min (3 or more times/ week)

**3. During the past month, what time have you usually gotten up in the morning?**

a) Before 06:00 am b) 06:00 - 06:59 am c) 07:00 - 07:59 am d) 08:00 - 08:59 am e) After 09:00 am

**4. During the past month, how many hours of actual sleep did you get at night? (This may be different than the number of hours you spent in bed.)**

a) < 7 hours b) > 7 hours

**Table:**

5. During the past month, how often have you had trouble sleeping because you...	Not during the past month	Less than once a week	Once or twice a week	Three or more times a week
a. Cannot get to sleep within 30 minutes				
b. Wake up in the middle of the night or early				
c. Have to get up to use the bathroom				
d. Cannot breathe comfortably				
e. Cough or snore loudly				
f. Feel too cold				
g. Feel too hot				
h. Have bad dreams				
i. Have pain				
j. Other reason(s), please describe:				

**Table:**

	Not during the past month	Less than once a week	Once or twice a week	Three or more times a week
**6. During the past month, how often have you taken medicine to help you sleep (prescribed or ‘’over the counter’’?**				
**7. During the past month, how often have you had trouble staying awake while driving, eating meals or engaging in social activity?**				

**Table:**

	No problem at all	Only a very slight problem	Somewhat of a problem	A very big problem
**8. During the past month, how much of a problem has it been for you to keep up enough enthusiasm to get things done?**				

**Table:**

	Very good	Fairly good	Fairly bad	Very bad
**9. During the past month, how would you rate your sleep quality overall?**				

**Table:**

	No bed partner or room mate	Partner/room mate in other room	Partner in same room but not same bed	Partner in same bed
**10. Do you have a bed partner or room mate?**				

**Table:**

	Not during the past month	Less than once a week	Once or twice a week	Three or more times a week
If you have a room mate or bed partner, ask him/her how often in the past month you have had:				
a. Loud snoring				
b. Long pauses between breaths while asleep				
c. Legs twitching or jerking while you sleep				
d. Episodes of disorientation or confusion during sleep				
e. Other restlessness while you sleep, please describe:				

**Scoring the PSQI**

The order of the PSQI items has been modified from the original order in order to fit the first 9 items (which are the only items that contribute to the total score) on a single page. Item 10, which is the second page of the scale, does not contribute to the PSQI score.

In scoring the PSQI, seven component scores are derived, each scored 0 (no difficulty) to 3 (severe difficulty). The component scores are summed to produce a global score (range 0 to 21). Higher scores indicate worse sleep quality.

**Component 1: Subjective sleep quality—question 9**

**Table:**

Response to Q9	Component 1 score
Very good	0
Fairly good	1
Fairly bad	2
Very bad	3

Component 1 score:_____

**Component 2: Sleep latency—questions 2 and 5a**

**Table:**

Response to Q2	Component 2/Q2 subscore
< 15 minutes	0
16-30 minutes	1
31-60 minutes	2
> 60 minutes	3
Response to Q5a	Component 2/Q5a subscore
Not during past month	0
Less than once a week	1
Once or twice a week	2
Three or more times a week	3
Sum of Q2 and Q5a subscores	Component 2 score
0	
1-2	1
3-4	2
5-6	3

Component 2 score:_____

**Component 3: Sleep duration—question 4**

**Table:**

Response to Q4	Component 3 score
> 7 hours	0
6-7 hours	1
5-6 hours	2
< 5 hours	3

Component 3 score:_____

**Component 4: Sleep efficiency—questions 1, 3, and 4**

Sleep efficiency = (# hours slept/# hours in bed) X 100% # hours slept—question 4

# hours in bed—calculated from responses to questions 1 and 3

**Table:**

Sleep efficiency	Component 4 score
> 85%	0
75-84%	1
65-74%	2
< 65%	3

Component 4 score:_____

**Component 5: Sleep disturbance—questions 5b-5j**

Questions 5b to 5j should be scored as follows:

**Table:**

Not during past month	0
Less than once a week	1
Once or twice a week	2
Three or more times a week	3
Sum of 5b to 5j scores	Component 5 score
0	0
1-9	1
10-18	2
19-27	3

Component 5 score:_____

**Component 6: Use of sleep medication—question 6**

**Table:**

Response to Q6	Component 6 score
Not during past month	0
Less than once a week	1
Once or twice a week	2
Three or more times a week	3

Component 6 score:_____

**Component 7: Daytime dysfunction—questions 7 and 8**

**Table:**

Response to Q7	Component 7/Q7 subscore
Not during past month	0
Less than once a week	1
Once or twice a week	2
Three or more times a week	3
Response to Q8	Component 7/Q8 subscore
No problem at all	0
Only a very slight problem	1
Somewhat of a problem	2
A very big problem	3
Sum of Q7 and Q8 subscores	Component 7 score
0	0
1-2	1
3-4	2
5-6	3
Component 7 score:_______________________________	

Component 7 score:_____

Global PSQI Score: Sum of seven component scores:_____________________________________________________________________________________

Copyright notice: The Pittsburgh Sleep Quality Index (PSQI) is copyrighted by Daniel J. Buysse, M.D. Permission has been granted to reproduce the scale on this website for clinicians to use in their practice and for researchers to use in non-industry studies. For other uses of the scale, the owner of the copyright should be contacted.

Citation: Buysse, DJ, Reynolds CF, Monk TH, Berman SR, Kupfer DJ: The Pittsburgh Sleep Quality Index (PSQI): A new instrument for psychiatric research and practice. Psychiatry Research 28:193-213, 1989
